# Internal and External Influences on Stability and Ligand Exchange Reactions in Bromido[3-ethyl-4-aryl-5-(2-methoxypyridin-5-yl)-1-propyl-1,3-dihydro-2*H*-imidazol-2-ylidene]gold(I) Complexes

**DOI:** 10.1021/acs.inorgchem.1c00325

**Published:** 2021-06-07

**Authors:** Sina Katharina Goetzfried, Sophie Marie Charlotte Koenig, Caroline Marie Gallati, Ronald Gust

**Affiliations:** Department of Pharmaceutical Chemistry, Institute of Pharmacy, Center for Molecular Biosciences Innsbruck, University of Innsbruck, Innrain 80/82, Innsbruck A-6020, Austria

## Abstract

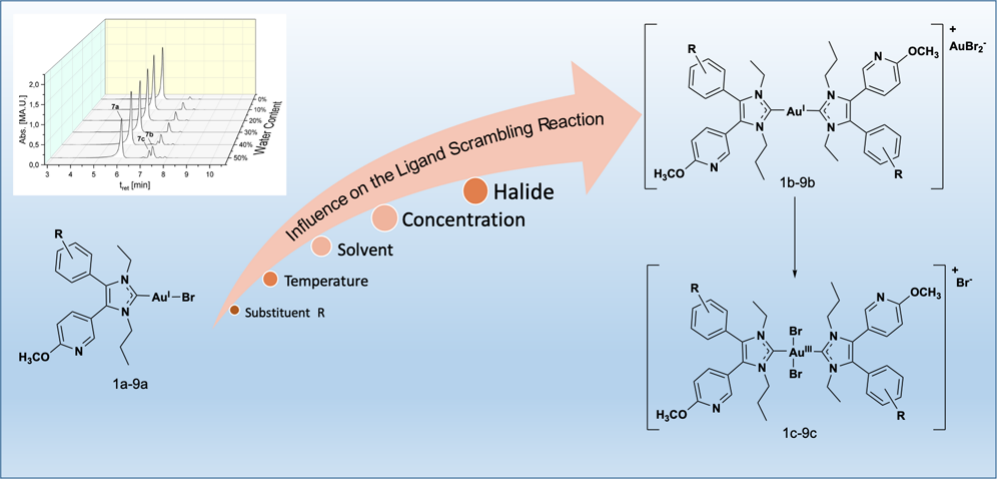

The ligand scrambling reaction of gold(I) complexes is a phenomenon occurring primarily in L–Au^I^–X (L = phosphine, *N*-heterocyclic carbene (NHC), and thiol; X = halide and thiol) complexes and has been observed among others for e.g., the bromido[3-ethyl-4-(4-methoxyphenyl)-5-(2-methoxypyridin-5-yl)-1-propyl-1,3-dihydro-2*H*-imidazol-2-ylidene]gold(I) complex (**7a**), which underwent ligand rearrangement in aqueous solutions. In this study, we investigated the influence of substituents on the 4-aryl ring of the related (NHC)Au^I^Br complexes (**1a**–**9a**) in terms of the conversion to the [(NHC)_2_Au^I^]^+^ (**1b**–**9b**) and [(NHC)_2_Au^III^Br_2_]^+^ (**1c**–**9c**) species. Furthermore, the influence of external factors such as solvent, temperature, concentration, and presence of halides (Cl^–^, Br^–^, and I^–^) or hydroxyl ions was studied to gain a deeper understanding of the ligand rearrangement reaction. The substituent on the 4-aryl ring has a marginal impact on the scrambling reaction. Out of the investigated organic solvents (dimethylformamide (DMF), dimethyl sulfoxide (DMSO), ethanol (EtOH), methanol (MeOH), and acetonitrile (ACN)), only ACN separates single complex molecules. In all other solvents, relatively stable ((NHC)Au^I^Br)_2_ dimers are present. The addition of water to ACN solutions forces the formation of such dimeric units, starting the transformation to [(NHC)_2_Au^I^]^+^ and [(NHC)_2_Au^III^Br_2_]^+^. The rate-determining step is the release of Br^–^ from a T-shape intermediate because an excess of KBr terminates this reaction. Furthermore, it is obvious that only single molecules react with halides. The aurophilic interactions between two (NHC)Au^I^Br molecules are too strong in the presence of water and largely impeded reaction with halides. As a single molecule, the reaction with Cl^–^ (e.g., in a 0.9% NaCl solution) is notable, while I^–^ even leads to a fast and quantitative conversion to (NHC)Au^I^I and finally to [(NHC)_2_Au^I^]^+^.

## Abstract 2

Internal and external factors of the ligand scrambling reaction in (NHC)Au^I^Br complexes were investigated by the means of HPLC. The data represent the impact of substituents, temperature, solvent, concentration and addition of halides on the conversion to the respective [(NHC)_2_Au^I^]^+^ and the oxidation to the [(NHC)_2_Au^III^Br_2_]^+^ complexes.

## Introduction

Ligand exchange reactions of metal complexes are a phenomenon with increasing interest in the scientific community.^1−8^ It is well known that rhodium, ruthenium, platinum, and gold complexes suffer ligand replacement reactions.^2,6,8^ For instance, cisplatin has to hydrolyze to reactive aqua species^9−12^ prior to binding to its targets.^2,13−19^ Additionally, ligand exchange is used to coordinate metallodrugs, e.g., gold complexes, to carrier ligands for high accumulation within cells.^18,20−23^

The stability of metal complexes depends on the central ion and the used ligands. Complexes of Mg^2+^, Ca^2+^, K^+^, and Na^+^ are less stable than those of transition metal ions and exchange the ligands rapidly. In contrast, Ru^2+^, Os^2+^, Ir^3+^, and Pt^2+/4+^ complexes require hours or even days.^24,25^

Gold(I) complexes are also susceptible to undergo rearrangement reactions.^4,6−8^ Ligands can be categorized as carrier ligands or leaving groups, depending on their binding strength to the metal. Suitable carriers represent *N*-heterocyclic carbenes (NHCs). Resulting (NHC)gold(I) complexes are promising candidates for the application in medicinal and inorganic chemistry because of their anticancer activity^26−29^ as well as luminescence^30−32^ and catalytic^33^ properties.

NHCs form strong σ-donor bonds to a number of metals,^34^ and the electronic factors stabilize the NHC-metal bond in a push–pull mechanism as a result of the σ- and π-frameworks.^35,36^ The electron-donor effects are higher than those of phosphines^37^ and the (NHC)gold(I) complexes are regarded to be stable in solution under standard conditions (room temperature (rt), protection from light, and dry atmosphere). Nevertheless, ligand exchange reactions are observed for a variety of complexes.^38,39^ Of high interest is the stability in the presence of water because it allows the estimation of their behavior under physiological conditions.

In a previous paper, we reported on the ligand scrambling of bromido[3-ethyl-4-(4-methoxyphenyl)-5-(2-methoxypyridin-5-yl)-1-propyl-1,3-dihydro-2*H*-imidazol-2-ylidene]gold(I) (**7a**). It was possible to identify intermediates in acetonitrile (ACN)/water (50:50, v/v) mixtures by high-performance liquid chromatography (HPLC) and high-resolution mass spectrometry (HR-MS), which made the suggestion of a plausible mechanism of ligand exchange possible.^40^

**Scheme 1:**
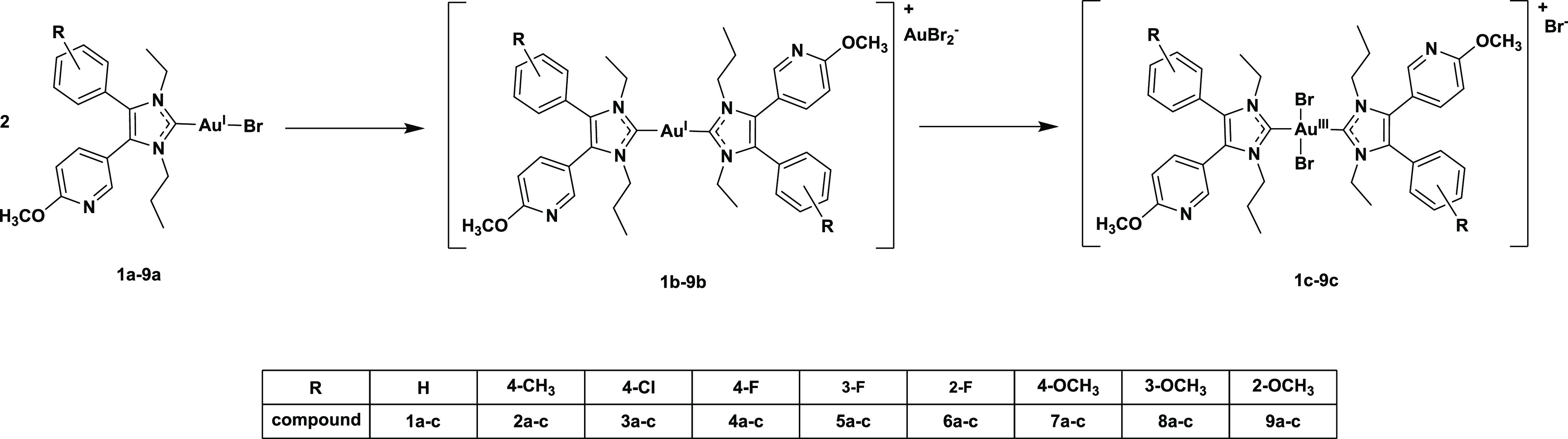
General Ligand Scrambling Reaction of **1a**–**9a** to **1b**–**9b** and the Subsequent Oxidation to **1c**–**9c**

In continuation, we studied parameters (substituents on the 4-aryl ring, variation of solvent, concentration, and temperature) that influence the conversion of (NHC)Au^I^Br complexes (**1a**–**9a**; Scheme 1) to the respective [(NHC)_2_Au^I^]^+^ (**1b**–**9b**) and [(NHC)_2_Au^III^Br_2_]^+^ species (**1c**–**9c**). The transformation was followed by HPLC because this method enabled quantitative analyses of the degradation products.

The data are important for the interpretation of the in vitro results since **1a**–**9a** exhibited antiproliferative activity in ovarian cancer and leukemia cell lines at low micromolar concentrations,^41^ whereas **1b**–**9b** caused these effects already at nanomolar concentrations.^42^ Therefore, it is of interest to know more about the conditions for the transformation of **1a**–**9a** to the higher active **1b**–**9b**. Furthermore, the reaction with chloride is pivotal because of the high concentration in the cell culture medium, which leads to the formation of (NHC)Au^I^Cl complexes. Thus, we investigated the stability of (NHC)Au^I^Br complexes in the presence of 0.9% NaCl on the examples of **7a** and **8a**.

In a second approach, the ligand exchange reactions in **8a** were studied using Cl^–^, Br^–^, I^–^, and OH^–^ as model nucleophiles. Such information is of relevance to estimate the reactivity of (NHC)Au^I^Br complexes against bionucleophiles.

## Results

### Sample Preparation

A solution of **1a**–**9a** (1 mM) in an appropriate mixture of ACN and water was monitored by HPLC for 72 h. The complexes were dissolved in ACN (or other organic solvents) and then diluted with water. The samples were filtered through a 0.20 μm membrane filter and analyzed after various incubation times (0, 24, 48, and 72 h) in HPLC experiments using the Shimadzu Prominence HPLC system equipped with a SIL-20A HT autosampler, a CTO-10AS VP column oven, a DGU-20A degasser, an SPD-M20A detector, LC-20AD pumps, and a KNAUER 250 × 4 nm^2^ Eurospher 100-C18 column. The mobile phase consisted of ACN and water with 0.1% trifluoroacetic acid (TFA). Separation of the complexes from the reaction product was possible with gradient elution from 70 to 90% ACN and a flow rate of 1 mL/min at an oven temperature of 35 °C. All solvents were degassed before use. The injection volume was 20 μL, and UV–vis detection was performed at 254 nm. Each measurement was performed in triplicates. Three-dimensional (3D) graphs of HPLC chromatograms were prepared using OriginPro 2016 (Northampton, MA). The peaks were assigned by the analysis of their UV–vis spectra or comparison with synthesized reference compounds ((NHC)Au^I^X; X = I: **8d**, X = Cl: **8e**, X = OH: **8f**).^41^

### Internal Influences

#### Substituent Effects

To study the influence of the substituents on the 4-aryl ring, the bromido (NHC)gold(I) complexes **1a**–**9a** (1 mM), each dissolved in ACN/water (50:50, v/v), were analyzed by HPLC for their degradation profile during 72 h of incubation at rt.

The amount of water determined the ligand scrambling of (NHC)Au^I^Br and the following oxidation of the resulting [(NHC)_2_Au^I^]^+^ to [(NHC)_2_Au^III^Br_2_]^+^ (Scheme 1). The highest degradation caused a 50% portion of water.^40^ Therefore, **1a**–**9a** were investigated under these conditions. The results are depicted in Figure 1 and data are listed in Table 1 (see also Figures S3–S11 and Table S1 in the Supporting Information).

**Figure 1:**
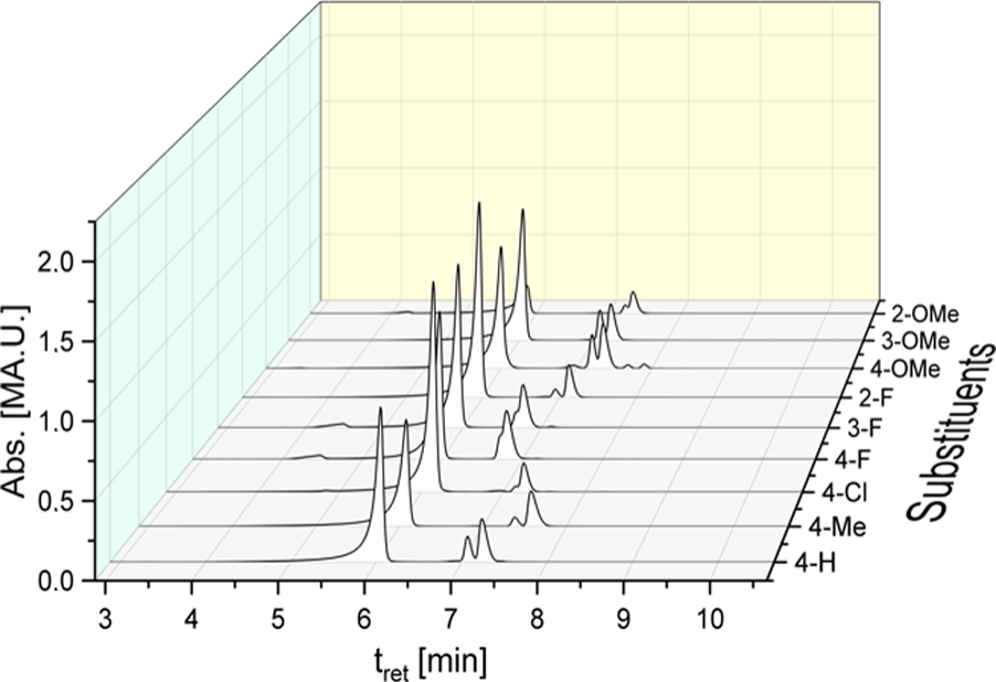
HPLC chromatograms of (NHC)Au^I^Br complexes (**1a–9a**) after 72 h of incubation in ACN/water mixture (50:50, v/v) at rt. First peak: (NHC)Au^I^Br; second peak: [(NHC)_2_Au^III^Br_2_]^+^; third peak: [(NHC)_2_Au^I^]^+^.

**Table 1:** Time-Dependent Degradation of (NHC)Au^I^Br Complexes (**1a–9a**) in ACN/Water (50:50, v/v)^a^

	(NHC)Au^I^Br (**1a–9a**) [%]		[(NHC)_2_Au^I^]^+^ (**1b–9b**) [%]		[(NHC)_2_Au^III^Br_2_]^+^ (**1c–9c**) [%]	
compound	24 h	72 h	24 h	72 h	24 h	72 h
**1**	88.68	83.15	10.43	11.09	0.88	5.75
**2**	89.24	78.69	10.42	18.13	0.34	3.17
**3**	94.85	90.51	4.96	9.02	0.18	0.47
**4**	85.55	74.12	14.45	20.23		5.65
**5**	89.35	78.56	9.75	17.62	0.88	3.82
**6**	92.75	88.14	7.06	10.22	0.17	1.64
**7**	84.62	75.72	12.67	13.95	1.50	8.19
**8**	89.73	80.58	9.59	11.75	0.67	7.66
**9**	81.13	68.72	17.03	24.30	1.84	6.97

a Analyzed by HPLC: gradient elution from 70 to 90% ACN in ACN/water (0.1% TFA) at a flow rate of 1 mL/min and an oven temperature of 35 °C; UV−vis detection at 254 nm.

Most complexes degraded in the range of 10–20% during 24 h. Only **3a** and **6a** showed higher stability (5.15 and 7.25% degradation, respectively). Through 72 h of incubation, the peak areas of the initial peaks (**1a**–**9a**) further decreased in the HPLC chromatograms. **9a** underwent the highest transformation, with 24.30% to **9b** and 6.97% to **9c**. It should be mentioned that the solubility of **9a** in ACN/water (50:50, v/v) was too low to realize a 1 mM solution. Therefore, the results must be handled with care.

The data listed in Table 1 indicate only a marginal influence of the substitution pattern of the 4-aryl ring on the complex stability. The introduction of a 2-OCH_3_ substituent (**9a**) accelerated the scrambling reaction, while 4-Cl (**3a**) and 2-F (**6a**) substituents caused the opposite effect. Nearly identical proportions of the [(NHC)_2_Au^I^]^+^ species (9–14%) were detected for **1**, **3**, **6**, **7**, and **8** after 72 h. Higher amounts were found for **2** (18.13%), **4** (20.23%), **5** (17.62%), **9** (24.30%). Oxidation to [(NHC)_2_Au^III^Br_2_]^+^ was observed in each case. After 72 h, portions higher than 5% were noticed for **1c** (5.75%), **4c** (5.65%), **7c** (8.19%), **8c** (7.66%), and **9c** (6.97%).

#### External Influences

For extended investigations on the dependence of external parameters on the ligand scrambling, complexes **7a** and **8a** were selected because they showed the best physicochemical properties, especially solubility in various solvents, and high resolution of the degradation products in the HPLC chromatograms.

#### Solvent Effects

**Figure 2:**
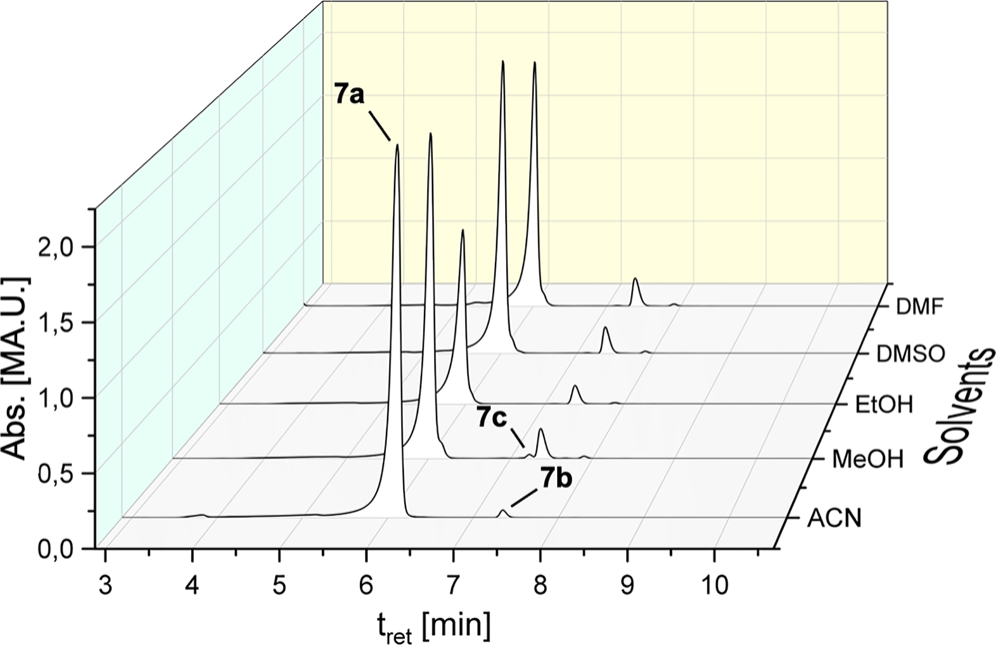
HPLC chromatogram of **7a** in water-free solvents after incubation for 72 h at rt.

To determine the influence of organic solvents on the ligand scrambling, 1 mM solutions of complex **7a** in water-free ACN, dimethylformamide (DMF), dimethyl sulfoxide (DMSO), ethanol (EtOH), and methanol (MeOH) were prepared and analyzed by HPLC (Figures 2, S12, S14, S16, S18, and S20 and Table S2 in the Supporting Information).

In ACN, the transformation to **7b** (2.75% at *t* = 0 h) was suppressed for 72 h (2.85%), while it increased in other solvents after 72 h by 2–3% (Table S2 in the Supporting Information). It is noteworthy that the solubility of **7a** was insufficient in EtOH (<1 mM), indicated by the reduced area of the main peak (Figures 2 and S18 (Supporting Information)).

The addition of water to the respective solutions of **7a** decreased the solubility. The complex was insufficiently soluble in DMF/water (50:50, v/v) (Figure S15 in the Supporting Information), while in the case of the other mixtures, the target concentration of 1 mM could be realized (*t* = 0 h), but the peak area in the HPLC chromatograms (Figures S17, S19, and S21 in the Supporting Information) drastically decreased within 24 h. Only in ACN/water (Figure S13 in the Supporting Information), sufficient solubility was guaranteed.

#### Concentration and Temperature Dependence

The dependence of the scrambling reaction on the concentration was studied in ACN/water (50:50, v/v). Table 2 lists the results obtained with **8a** at 0.5, 1, and 2 mM after an incubation time of 72 h at rt. The relevant HPLC chromatograms are depicted in Figures S22–S24 (Supporting Information). The data at *t* = 0, 24, and 48 h are listed in Table S3 (Supporting Information).

**Table 2:** Transformation of **8a** in ACN/Water (50:50, v/v) at Different Concentrations and Various Temperatures (Conc. 1 mM) after 72 h of Incubation^a^

		**8a**	**8b**	**8c**
concentration	0.5 mM	86.27	8.65	5.07
	1 mM	80.58	11.74	7.66
	2 mM	76.17	15.42	8.42
temperature	4 °C	84.69	12.96	2.33
	22 °C	80.58	11.74	7.66
	37 °C	59.77	27.89	12.33
	50 °C	65.20	25.94	8.85
	80 °C	57.84	42.15	

a See Table 1 for HPLC conditions.

The transformation of **8a** to the [(NHC)_2_Au^I^]^+^ species **8b** and the oxidized [(NHC)_2_Au^III^Br_2_]^+^ form **8c** increased with concentration. At 0.5 mM, the proportion of **8a** amounted to 86.27%, at 1 mM to 80.58%, and at 2 mM to 76.17%. Unfortunately, the concentrations could not be reduced to those relevant for in vitro studies. The sensitivity of the HPLC limited the analysis at concentrations < 50 μM.

The solution of complex **8a** in ACN/water (50:50, v/v) was further investigated after incubation at various temperatures (4, 22, 37, 50, and 80 °C) for 72 h (Table 2). Cooling to 4 °C reduced the ligand scrambling: 84.69% of **8a** remained unchanged, while 12.96% of **8b** as well as 2.33% of **8c** were detected. Compared to the incubation at 22 °C, mainly the amount of **8c** was reduced. In contrast, incubation at 37 °C strongly increased the transformation to **8b** (27.89%) and especially **8c** (12.33%).

**Figure 3:**
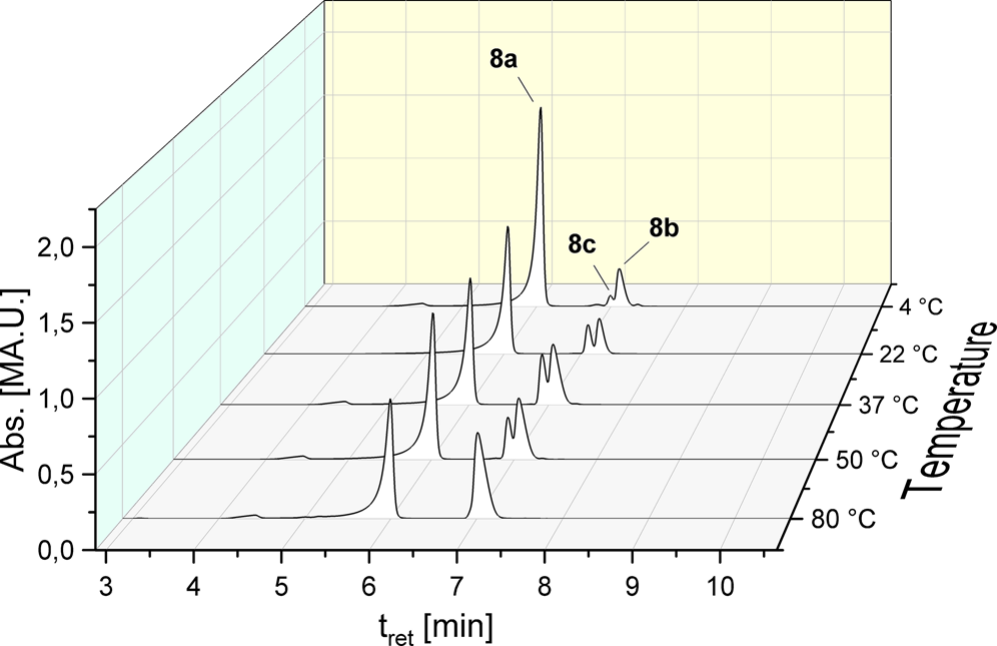
HPLC chromatograms of **8a** after incubation in ACN/water (50:50, v/v) at different temperatures for 72 h.

Interestingly, a further increase in the temperature reduced the oxidation to **8c**. At 50 °C, the percentage distribution was **8a**: 65.20%, **8b**: 25.84%, and **8c**: 8.85%. At 80 °C, no oxidized species **8c** was detectable. **8a** only transformed to **8b** in a ratio of 42.15% (Figure 3).

#### Effect of KCl, KBr, KI, and KOH Addition

Besides ligand scrambling reactions, the exchange of the bromido ligand in gold(I) is possible. Especially, the reactions with other halides are of interest. Therefore, **7a** and **8a** solutions (1 mM) in ACN/water (50:50, v/v) were incubated with an excess of KCl, KBr, or KI (20 equiv each) for 72 h at rt.

**Figure 4:**
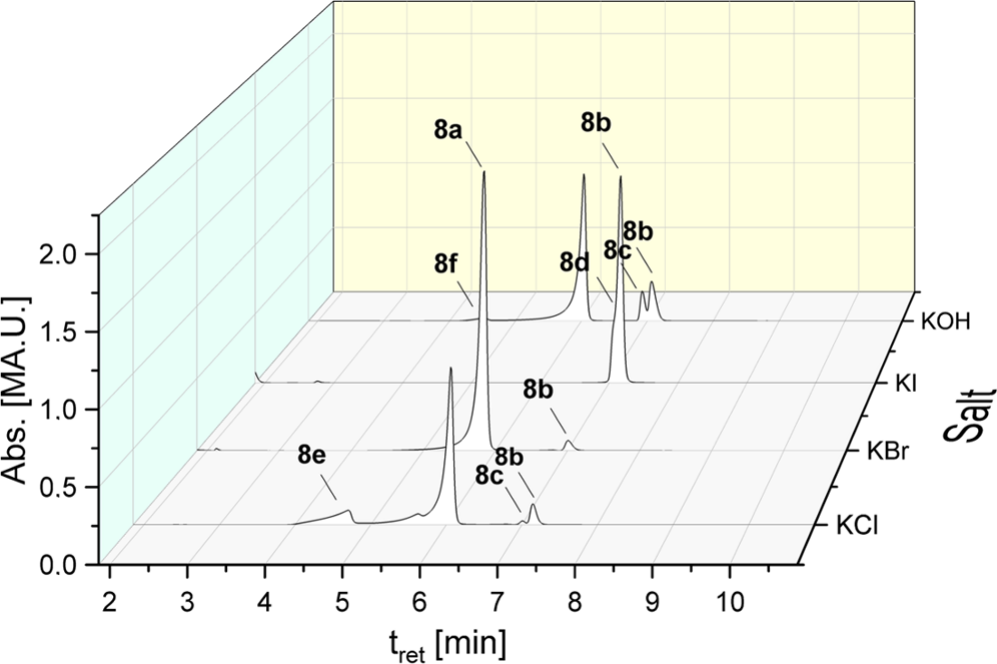
HPLC chromatograms of **8a** in ACN/water (50:50, v/v; with 20 equiv of KCl, KBr, KI, or KOH) after 72 h of incubation at 22 °C.

Figure 4 illustrates the chromatograms (*t* = 72 h) exemplarily for **8a**. Analytical data for **8a** are listed in Table 3 (for **7a**, see Table S4 and Figures S25–S28 in the Supporting Information).

**Table 3:** Reaction of **8a**–**8c** to (NHC)Au^I^X Complexes **8x** (X = I: x = d; X = Cl: x = e; X = OH: x = f) after an Incubation Time of 72 h at 22 °C^a^

	**8a**	**8b**	**8x**	**8c**
KI		76.49	23.05	
KBr	97.53	2.46		
KOH	73.18	15.86	2.00	8.95
KCl	77.56	6.39	15.44	

a See Table 1 for HPLC conditions.

KBr stabilized **8a** and the amount of **8b** remained unchanged during the incubation for 72 h at 22 °C (*t* = 0 h: 2.31%; *t* = 72 h: 2.46%; Table S4 in the Supporting Information). Without KBr, degradation of **8a** strongly increased (**8b:** 11.75%; **8c**: 7.66%; Table 1). An opposite effect was observed upon addition of KI. The peak of **8a** completely disappeared and **8b** was built as the main peak (76.49%). (NHC)Au^I^I complex **8d** was visible as a small shoulder. In contrast, only 15.44% of **8a** reacted immediately after dissolution with chloride (KCl) to (NHC)Au^I^Cl (**8e**). This proportion remained constant for 72 h. Increased amounts of **8b** and **8c** resulted from degradation of remaining **8a**.

The hydroxide ion (KOH) was used as another nucleophile. It reacted with **8a** only in small amounts (2.0%) to form (NHC)Au^I^OH (**8f**) upon dissolution. The ligand scrambling reaction was nearly independent of the presence of KOH. After 72 h, 15.86% of **8b** (11.75% without KOH) and 8.95% of **8c** (7.66% without KOH) were detected.

Finally, it is of interest to know the extent of Br^–^/Cl^–^ exchange under physiological NaCl conditions. For this purpose, the most active complex **7a**(41) was dissolved in ACN and a 1.8% NaCl solution was added to obtain a 50:50 (v/v) mixture with 0.9% NaCl at a complex concentration of 1 mM. In agreement with the above-described results, 34.27% of **7a** reacted initially (*t* = 0 h) to (NHC)Au^I^Cl (**7e**) (Table S7 and Figure S38 in the Supporting Information). Besides, the chromatogram exhibited peaks of **7a** (58.06%) and **7b** (7.16%). While the amount of **7e** remained unchanged (33.46%) for 72 h, about 5% of **7a** was transformed to **7b** (Table S7 in the Supporting Information). Complex **7b** was investigated in the same way and proved to be stable in the NaCl solution (Figure S39 in the Supporting Information) and no oxidation to **7c** took place.

## Discussion

In a previously published study, we described the ligand scrambling of the bromido (NHC)gold(I) complex **7a** to the [(NHC)_2_Au^I^]^+^ species (**7b**) followed by oxidation to [(NHC)_2_Au^III^Br_2_]^+^ (**7c**).^40^ In this paper, we confirmed this transformation for a series of 4-aryl-substituted derivatives (H, 4-CH_3_, 4-Cl, 4-F, 3-F, 2-F, 4-OCH_3_, 3-OCH_3_, and 2-OCH_3_) in ACN/water (50:50, v/v) solutions. The influence of the 4-aryl ring substituents, however, was only marginal. Stability mediated the 4-Cl (**3a**) and 2-F (**6a**) substitution, while the 2-OCH_3_ (**9a**) substituent caused more degradation. All degradation products can be sufficiently detected and separated by HPLC (Figure 2).

Effects of the substituents on the C–Au^I^Br bond can be excluded because N–C–N resonances in the ^13^C NMR spectra (CDCl_3_) are nearly identical in the range of 173.3–174.6 ppm, indicating a comparable influence on the strength of the Au^I^–Br bond.

The solubility of **7a** in dry ACN, DMF, DMSO, and MeOH allowed the preparation of 1 mM stock solutions. Only in EtOH, this concentration could not be realized. In aqueous mixtures of these solvents, the solubility was strongly reduced. Indeed, it was possible to achieve (with exception of DMF) initially 1 mM concentrations in 50:50 (v/v) mixtures, but **7a** crystallized during the incubation for 72 h, indicated by a strong reduction of the peak area in the chromatograms (Figures S15, S17, S19, and S21 in the Supporting Information). The X-ray analysis of the precipitate confirmed the presence of **7a** dimers with strong aurophilic bonds.^41^ Hence, it can be deduced that the higher polarity of the solvents/water mixtures forces the formation of these “hydrophobic” gold(I)–gold(I) interactions in solution, which is accompanied by lower solubility than under water-free conditions. Furthermore, upon the building of the dimers, ligand scrambling took place and the peak area of the [(NHC)_2_Au^I^]^+^ species increased to 8–10% related to the initial area of the (NHC)Au^I^Br peak.

It was further documented that dry ACN stabilizes (NHC)Au^I^Br complexes as monomers in solution, preventing degradation. In DMF, DMSO, EtOH, and MeOH, the portion of the [(NHC)_2_Au^I^]^+^ complex increased within 72 h by 2–3%. Therefore, it can be assumed that dimer formation through aurophilic interactions is possible and these organic solvents are inappropriate for the preparation of stock solutions and long-time storage. Unfortunately, most scientists use DMSO and DMF as solvents for in vitro experiments of gold(I) complexes. An increased transformation to the more active [(NHC)_2_Au^I^]^+^ species upon storage is possible and has to be excluded prior to the start of the experiments because it sophisticates the collected data.

**Scheme 2:**
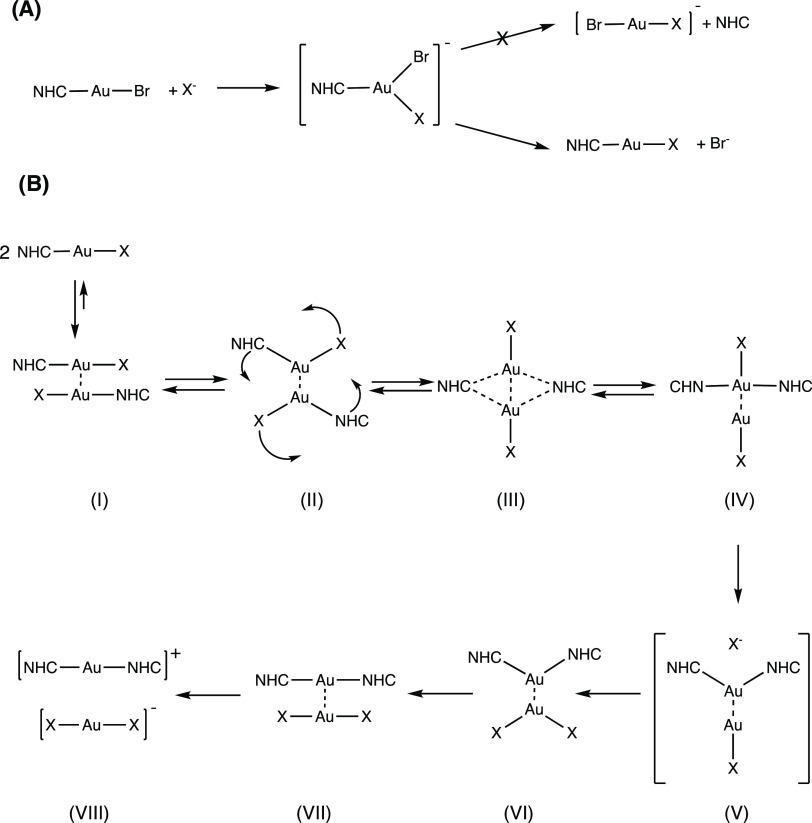
Schematic Drawing of (A) Substitution Reaction of (NHC)Au^I^Br Complexes and (B) Proposed Mechanism of the Ligand Scrambling between Two (NHC)Au^I^Br Complexes

We already presented a plausible mechanism for the ligand scrambling,^40^ starting from dimeric (NHC)Au^I^Br adducts, stabilized by strong gold(I)–gold(I) interactions,^43−46^ originating from the lanthanide contraction and the relativistic effects in gold.^35,47,48^ A simplified schematic drawing is depicted in Scheme 2B.

To gain a better knowledge about the conditions for this reaction, we investigated its dependence on concentration and temperature in the ACN/water mixture (50:50, v/v) on the example of **8a**. The proportion of the [(NHC)_2_Au^I^]^+^ (**8b**) and [(NHC)_2_Au^III^Br_2_]^+^ (**8c**) species increased with the concentration. After 72 h of incubation at rt, 86.27% of **8a** remained unchanged at 0.5 mM, 80.58% at 1 mM, and only 76.17% at 2 mM. Thus, it can be assumed that higher concentrations forced the aurophilic interactions.

Furthermore, the degradation depended on the temperature. Cooling to 4 °C reduced the oxidation to **8c** (7.66% (rt) → 2.33%), while the amount of **8b** was nearly constant (11.74% (rt) → 12.96%) after 72 h. Higher temperatures, e.g., 37 and 50 °C, increased the content of **8b** to 26–28%. Interestingly, the oxidation to **8c** decreased. These data clearly demonstrate that higher temperatures favor the intermolecular ligand exchange.

In the next step, it was of interest to study the reaction of **8a** with nucleophiles in ACN/water (50:50, v/v). For this purpose, the complex dissolved in ACN and water containing KX salts (X = Cl, Br, I) was added to achieve a 1 mM complex solution with 20 equiv excess of halide.

The attack of the nucleophile at the gold(I) center yields a trigonal intermediate. The complex can then either be stabilized by the release of NHC or by one of the bound halides (Scheme 2A). Liberation of the organic ligand can be excluded because it was never detected in the HPLC chromatograms.

The contact of **8a** with KCl in ACN/water caused already at *t* = 0 h the formation of 15.44% (NHC)Au^I^Cl **8e**. This ratio remained constant during the incubation for 72 h, while remaining **8a** degraded to **8b** and **8c**. This finding confirmed the initial presence of monomeric (NHC)Au^I^Br molecules in ACN. Only in this case, Br^–^/Cl^–^ exchange (Scheme 2A) is possible upon addition of the aqueous KCl solution. Immediately after the preparation of the mixture, aurophilic interactions cause the formation of dimers and intermediate II (Scheme 2B), which very likely prevents the simple halide exchange.

These reactions are principally also possible in the presence of KBr. However, it was observed that the excess of Br^–^ delays the ligand scrambling. We postulate the interference in successive reaction steps after dimer formation. Rate determining seems to be the dissociation of Br^–^ from the T-shape intermediate (IV), which is suppressed by the excess of Br^–^.

Iodide is a very interesting anion to study the reactivity of metal complexes. It represents on the one hand a strong nucleophile and on the other hand an excellent leaving group.^49^ The nucleophilic potency is comparable to that of guanosine and can therefore also be used to estimate the reactivity against bionucleophiles in a model reaction.

After combining the complex containing ACN with the aqueous KI solution, fast Br^–^/I^–^ exchange took place. Iodide reacted with **8a** giving **8d** in quantitative yield. Subsequently, [(NHC)Au^I^I]_2_ dimers are formed and rearranged to **8b**, forced by the excellent leaving group behavior of I^–^ (intermediate IV → V; Scheme 2). During the incubation of 72 h, the proportion of **8d** and **8b** remained nearly constant and no oxidized species was detected.

For the interpretation of the biological results, it is of importance to know more about the transformation of drugs in a 0.9% NaCl solution. The reaction of (NHC)Au^I^Br with biomolecules follows the pathway depicted in Scheme 2A. Possible Br^–^/Cl^–^ exchange at the gold(I) center prior to the binding to the target influences the biological outcome. In the 0.9% NaCl solution, bromido (NHC)gold(I) complex **7a** is subjected to about 35% transformation to the related chlorido (NHC)gold(I) complex **7e** immediately after dissolution. This proportion remained unchanged during the incubation for 72 h, while the amount of [(NHC)_2_Au^I^]^+^ complex **7b** increased from 7.16 to 12.16%. Therefore, **7a**, **7b**, and **7e** participated in the biological activity.

We already demonstrated that **7b** (IC_50_ = 0.26–0.63 μM) was about 8–10-fold more active than **7a** (IC_50_ = 3.0–6.5 μM) in various cell lines.^41^ Data about (NHC)Au^I^Cl complex **7e** is not available yet. However, it is well known that chlorido (NHC)gold(I) derivatives influence the growth of tumor cells less effectively than their leaving group derivatives. For instance, the IC_50_ values of the congeneric chlorido[3-ethyl-4-phenyl-5-(2-methoxypyridin-5-yl)-1-propyl-1,3-dihydro-2*H*-imidazol-2-ylidene]gold(I) complex were higher than 10 μM (data not shown). This finding is in accordance with the results of Rubbiani et al.^50,51^ They determined IC_50_ values between 5 and 10 μM for chlorido[1,3-diethylbenzylimidazol-2-ylidene]gold(I) complexes. The related [(NHC)_2_Au^I^]^+^ complexes were more active with IC_50_ = 0.4–0.9 μM. The same activity was observed for bis[1,3-diethyl-4,5-diaryl-1,3-dihydro-2*H*-imidazol-2-ylidne]gold(I) (IC_50_ = 0.2–0.5 μM)^52,53^ and bis[1,3-diethyl-4-aryl-1,3-dihydro-2*H*-imidazol-2-ylidne]gold(I) complexes (IC_50_ = 0.1–0.25 μM).^29,38,54^ The related chlorido (NHC)gold(I) derivatives were about 10-fold less active.

These data clearly point to a considerable role of the ligand (see also Tacke et al.^55,56^) that acts as a leaving group in the biological activity. Therefore, regarding the interpretation of the biological effects of (NHC)Au^I^Br complexes, the transformation under cell culture conditions must be considered. The complexes react within minutes with NaCl to less active chlorido (NHC)gold(I) complexes but also to bis(NHC)gold(I) species with higher activity. This degradation allows only an insufficient evaluation of the contribution of the bromido derivatives on the biological effects. Thus, it is necessary to examine the reactivity of (NHC)Au^I^X (X = halide, NHC) derivatives in more detail, immediately after dissolution in the cell culture medium and during the first 12 h of incubation, which is relevant to cellular accumulation. Such investigations will be part of a forthcoming paper.

## Conclusions

In this structure–activity relationship study, we investigated internal and external parameters essential for the ligand exchange reactions in bromido[3-ethyl-4-aryl-5-(2-methoxypyridin-5-yl)-1-propyl-1,3-dihydro-2*H*-imidazol-2-ylidene]gold(I) complexes. An increase in concentration and a temperature of 37 °C favor the formation of (NHC)Au^I^Br dimers, followed by ligand scrambling. The ligand exchange reaction as investigated with Cl^–^ and I^–^ occurs only with (NHC)Au^I^Br monomers (not with the [(NHC)_2_Au^I^]^+^ species), resulting in (NHC)Au^I^X (X = Cl, I) complexes. The presence of bromide renders the dissociation of Br^–^ from T-shape intermediate IV (Scheme 2B) and prevents the transformation to the [(NHC)_2_Au^I^]^+^ complex. The Br^–^/X^–^ exchange is of high relevance because it takes place at the moment when stock solutions (organic solvents) come in contact with aqueous media, e.g., used in in vitro assays. For the interpretation of the biological results, it must be considered that not only (NHC)Au^I^Br but also the formed (NHC)Au^I^Cl and [(NHC)_2_Au^I^]^+^ complexes participate in the observed effects. Therefore, we studied the solution behavior and the cytotoxicity of (NHC)Au^I^X (X = Cl, Br, I) complexes in more detail. The results are of interest for the interpretation of in vitro data and will be part of a forthcoming paper.

## Supporting Information Available

Materials and methods; syntheses; ^1^H and ^13^C spectra of complex **3a**; data (at *t* = 0–72 h) on the stability of **1a**–**9a** in ACN/water; stability data of **7a**/**8a** in ACN/water dependent on solvent, concentration, salt addition, and temperature; stability in 0.9% NaCl solutions; corresponding HPLC chromatograms; UV–vis spectra of the NHC ligand, **7a**, and **7b** (PDF)
