# Supplementary material for: Internal and External Influences on Stability and Ligand Exchange Reactions in Bromido[3-ethyl-4-aryl-5-(2-methoxypyridin-5-yl)-1-propyl-1,3-dihydro-2H-imidazol-2-ylidene]gold(I) Complexes
Source: Inorg Chem. 2021 Jun 7;60(12):8546–53. doi: 10.1021/acs.inorgchem.1c00325 (PMC8277168; doi:10.1021/acs.inorgchem.1c00325)
Supplement: Supplementary file 1 — ic1c00325_si_001.pdf [file ic1c00325_si_001.pdf]

Supporting Information

Internal and External Influences on Stability and Ligand Exchange Reactions in Bromido[3-ethyl-4-aryl-5-(2-methoxypyridin-5-yl)-1-propyl-1,3-dihydro-2*H*-imidazol-2-ylidene]gold(I) Complexes

Sina K. Goetzfried, Sophie M. C. Koenig, Caroline M. Gallati, Ronald Gust*

Dr. S. K. Goetzfried, Mag. S. M. C. Koenig, Dr. C. M. Gallati, Prof. Dr. R. Gust,

Department of Pharmaceutical Chemistry, Institute of Pharmacy, Center for Molecular Biosciences Innsbruck, University of Innsbruck, Innrain 80/82, 6020 Innsbruck, Austria.

E-mail: ronald.gust@uibk.ac.at

Table of contents

1. General Methods 2
2. Synthesis and Characterization 2
3. Influence of Substituents 4
4. Dependence on the Solvent 7
5. Concentration Dependence 9
6. Effect of KCl, KBr, KI or KOH Addition 10
7. Dependence on the Temperature 12
8. Stability in 0.9% NaCl 13
9. UV-Spectra of Compounds **NHC ligand**, **7a**, and **7b** 14

1. General Methods

Chemical reagents and solvents were purchased from commercial suppliers (Sigma-Aldrich, Fluka, Alfa Aesar and Acros) and were used without further purification. Analytical thin layer chromatography on silica gel was carried out on Polygram® SIL G/UV_{254} (Macherey-Nagel) plastic backed plates (0.25 mm layer thickness) with fluorescent indicator and Merck TLC Silica gel 60 F_{254} aluminium backed plates or Polygram® ALOX N/UV254 (Macherey-Nagel) plastic backed plates (0.2 mm layer thickness) with fluorescent indicator. The spots were visualised by UV light (254 nm and/or 365 nm). Column chromatography was performed using silica gel 60 (0.040-0.063 mm) or aluminium oxide 90 active neutral (Merck). Magnetic stirrer and heater: Heidolph MR 3001K/Heidolph EKT 3001. Evaporator: Heidolph Laborota 4000 efficient. Diaphragm pump: Ilmvac 600 T. Microwave assisted reactions were performed in a CEM Discover microwave (Software Synergy, CEM corporation). NMR spectra: Bruker Avance 4 Neo spectrometer (^{1}H: 400 MHz, ^{13}C: 100 MHz). The centre of the solvent signal and the TMS signal were used as internal standard. Deuterated solvents purchased from Euriso-top® were used as solvent for the samples. UV spectra were recorded on Thermo Fischer Scientific Multiscan Go with cuvette measurement.

HPLC-Studies

The HPLC experiments were performed with a Shimadzu prominence HPLC: auto sampler SIL-20A HT, column oven CTO-10AS VP, degassers DGU-20A, detector SPD-M20A, pumps LC-20AD, KNAUER 250 x 4nm Eurospher 100-5 C18 column. The mobile phase consisted of ACN and water with 0.1% TFA. To achieve a separation of the compounds the gradient elution with a composition of 70%-90% ACN was used with flow rate of 1 mL/min at an oven temperature of 35°C. All solvents were degassed before use. The injection volume was 20 µL and the gold complexes were detected at 254 nm. Each measurement was repeated four times and the average value as well as the standard deviation were calculated.

Each sample was freshly prepared. The powdered complexes were first dissolved in organic solvent and afterwards water was added to obtain a concentration of 1 mM in 1.50 mL. Sample solutions were passed through a 0.20 µm membrane filter.

2. Synthesis and Characterization

The syntheses of complexes **1**, **2**, **4-9** are described in Ref. 1 and 2.

Synthesis of complex 3a according to Ref 1.

***N*-(4-Chlorophenyl(tosyl)methyl)formamide**

Method B: colorless solid, 5670 mg from 3000 mg of 4-chlorobenzaldehyde (82 % yield). ^{1}H NMR (DMSO-d_6): δ = 2.41 (s, 3 H), 6.48 (d, 1 H, J = 10.4 Hz), 7.41-7.63 (m, 6 H), 7.74 (d, 2 H, J = 8.4 Hz), 7.97 (s, 1 H), 9.80 (d, 1 H, J = 10.4 Hz).

***N*-(1-(4-Chlorophenyl)-2-(6-methoxypyridin-3-yl)-2-*oxo*-ethyl)formamide**

Colorless oil, 1.50 g from 1.20 g of 6-methoxynicotinaldehyde (43 % yield). ^{1}H NMR ($CDCl_3$): δ = 3.97 (s, 3 H), 6.48 (d, 1 H, J = 7.0 Hz), 6.75 (d, 1 H, J = 8.8 Hz), 7.20-7.37 (m, 5 H), 8.11 (dd, 1 H, J = 8.8, 2.4 Hz), 8.26 (s, 1 H), 8.78 (d, 1 H; J = 2.4 Hz). ^{13}C NMR ($CDCl_3$): δ = 54.5, 56.7, 111.8, 123.7, 129.7, 129.8, 134.9, 135.4, 138.9, 150.5, 160.2, 167.4, 192.5.

5-(4-(4-Fluorophenyl)-1-propyl-1*H*-imidazol-5-yl)-2-methoxypyridine

Colorless oil, 632 mg from 1.092 g of *N*-(1-(4-chlorophenyl)-2-(6-methoxypyridin-3-yl)-2-*oxo*-ethyl)formamide (54 % yield). ^{1}H NMR ($CDCl_3$): δ = 0.86 (t, 3 H, *J* = 7.4 Hz), 1.63 (qt, 2 H, *J* = 7.4, 7.4 Hz), 3.75 (t, 2 H, *J* = 7.4 Hz), 4.01(s, 3 H), 6.85 (d, 1 H, *J* = 8.8 Hz), 7.19 (d, 2 H, *J* = 8.6 Hz), 7.40 (d, 2 H, *J* = 8.6 Hz), 7.50 (dd, 1 H, *J* = 8.8, 2.4 Hz), 7.63 (s, 1 H), 8.13 (d, 1 H, *J* = 2.4 Hz).^{13}C NMR ($CDCl_3$): δ = 11.2, 24.4, 47.0, 53.9, 111.7, 119.7, 125.3, 128.0, 128.6, 132.3, 133.1, 137.4, 138.4, 141.1, 148.7, 164.4.

3-Ethyl-4-(4-chlorophenyl)-5-(2-methoxypyridin-5-yl)-1-propyl-3*H*-imidazolium iodide

Colorless oil, 142 mg from 206 mg of imidazole 5-(4-chlorophenyl)-1-ethyl-4-(2-methoxypyridin-5-yl)-3-propyl-3*H*-imidazolium iodide (47 % yield).^{1}H NMR ($CDCl_3$): δ = 0.96 (t, 3 H, *J* = 7.4 Hz); 1,54 (t, 3 H, *J* = 7.4 Hz), 1.93 (qt, 2 H, *J* = 7.4, 7.4 Hz), 3.95 (s, 3 H), 4.15-4.35 (m, 4 H), 6.80 (d, 1 H, *J* = 8.6 Hz), 7.30 (d, 2 H, *J* = 8.4 Hz), 7.42 (d, 2 H, *J* = 8.2 Hz), 7.61 (dd, 1 H, *J* = 8.8, 2. 4 Hz), 8.07 (d, 1 H, *J* = 2.6 Hz), 10.41 (s, 1 H).^{13}C NMR ($CDCl_3$): δ = 11.1, 15.9, 23.7, 43.9, 49.7, 54.1, 112.1, 114.0, 123.2, 129.7, 130.0, 131.7, 132.1, 136.6, 137.3, 140.6, 148.9, 165.4.

3-Ethyl-4-(4-chlorophenyl)-5-(2-methoxypyridin-5-yl)-1-propyl-3*H*-imidazolium hexafluorophosphate

Off-white solid, 44 mg from 63 mg 5-(4-(4-chlorophenyl)-1-propyl-1*H*-imidazol-5-yl)-2-methoxypyridine (70 % yield).^{1}H NMR ($CDCl_3$): δ= 0.91 (t, 3 H, *J* = 7.4 Hz), 1.43 (t, 3 H, *J* = 7.2 Hz), 1.80 (qt, 2 H, *J* = 7.6, 7.6 Hz), 3.94 (s, 3 H), 4.00-4.19 (m, 4 H), 6.79 (d, 1 H, *J* = 8.6 Hz), 7.28 (d, 2 H, *J* = 7.2 Hz), 7.41 (d, 2 H, *J* = 8.6 Hz), 7.57 (dd, 1 H, *J* = 8.6, 2.4 Hz), 8.07 (d, 1 H, *J* = 2.2 Hz), 8.83 (s, 1 H).^{13}C NMR ($CDCl_3$): δ = 10.9, 14.9, 23.2, 43.6, 49.6, 54.0, 112.0, 114.2; 123.4, 129.9 (two signals overlapping), 132.0, 132.1, 135.4, 137.2, 140.6, 148.9. 165.3. ESI-MS m/z: 356.1514 ($[M-PF_6]^+$) Purity calculated by HPLC (peak area): 99.5 %

Bromido[3-ethyl-4-(4-chlorophenyl)-5-(6-methoxypyridin-3-yl)-1-propyl-1,3-dihydro-2*H*-imidazol-2-ylidene]gold(I) (3a)

Colorless solid, 36 mg from 35 mg of 3-ethyl-4-(4-chlorophenyl)-5-(2-methoxypyridin-5-yl)-1-propyl-3*H*-imidazolium hexafluorophosphate (82 % yield). ^{1}H NMR ($CDCl_3$): δ = 0.85 (t, 3 H, *J* = 7.4 Hz), 1.31 (t, 3 H, *J* = 7.2 Hz), 1.75 (qt, 2 H, *J* = 7.6, 7.6 Hz), 3.94 (s, 3 H), 4.07(q, 2 H, *J* = 7.6 Hz), 4.18 (q, 2 H, *J* = 7.2 Hz), 6.76 (d, 1 H; *J* = 8.4 Hz), 7.16 (d, 2 H, *J* = 8.80 Hz), 7.35-7.43 (m, 3 H), 7.99 (d, 1 H, *J* = 2.0 Hz). ^{13}C NMR ($CDCl_3$): δ = 11.3, 17.1, 24.9, 44.5, 50.9, 54.0, 111.6, 116.6, 125.9, 128.6, 129.6, 130.8, 131.9, 136.1, 140.3, 148.7, 164.7, 174.2. ESI-MS: m/z: 907.2518 $(2M-Au-Br_2)^+$, 1185.1332 $(2M-Br)^+$, 593.1384 $(M-Br+ACN)^+$. Purity: calculated by HPLC (peak area): 97.3 %

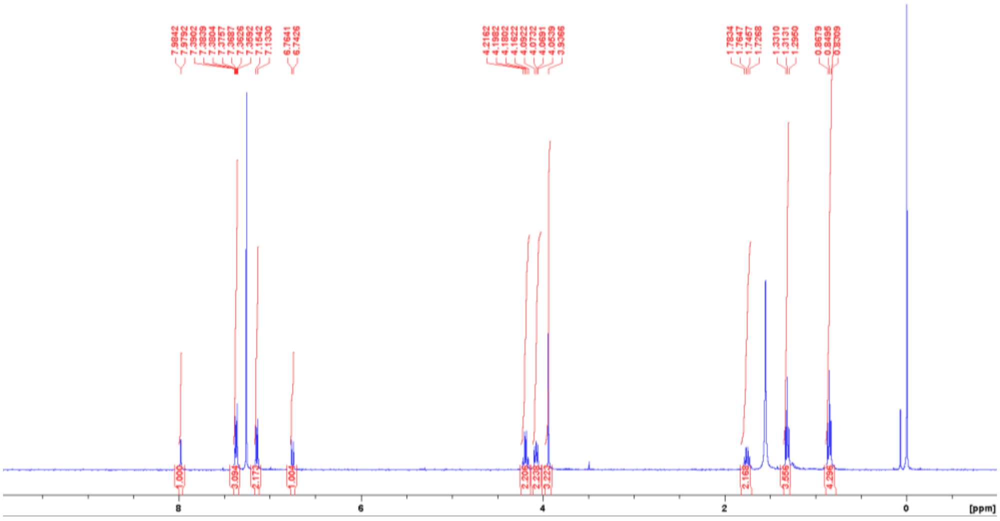

Figure S1. ^{1}H NMR spectrum of compound **3a** recorded in $CDCl_3$.at 400 MHz

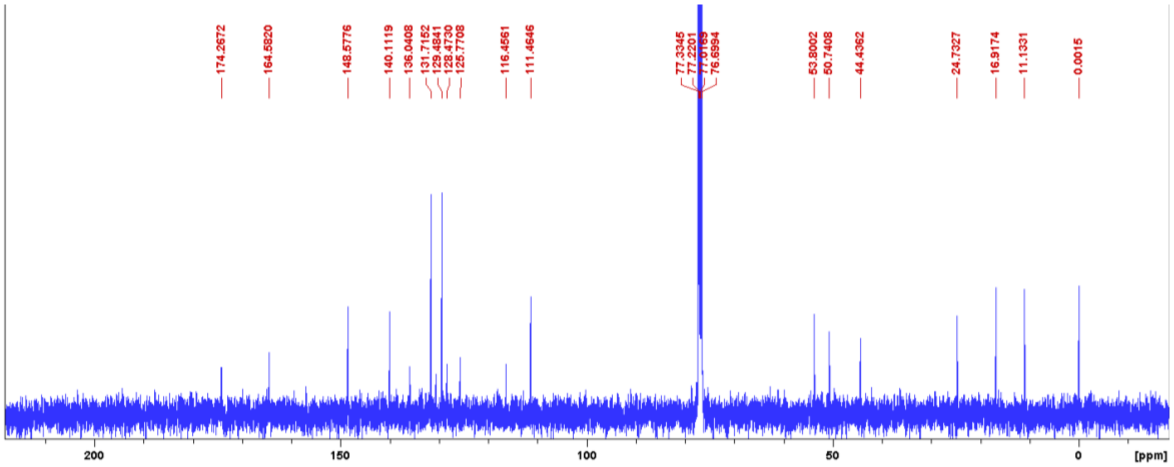

Figure S2. ^{13}C NMR spectrum of compound **3a** recorded in $CDCl_3$ at 100 MHz.

3. Influence of Substituents

Table S1. Average percentage values (AD [%]) and standard deviation (SD [%]) of mono-NHC gold(I) complexes 1a-9a and the resulting complexes 1b-9b and 1c-9c during incubation at rt in ACN/water (50:50, v/v).

	t = 0 h		*t = 24 h*		*t = 48 h*		*t = 72 h*	
	AD [%]	SD [%]	AD [%]	SD [%]	AD [%]	SD [%]	AD [%]	SD [%]
1a	96.54	0.13	88.68	0.37	84.08	0.40	83.15	6.08
1b	3.46	0.13	10.43	0.29	13.21	0.63	11.09	6.69
1c	-	-	0.88	0.08	2.70	0.34	5.75	1.11
2a	95.97	0.27	89.24	0.27	84.46	0.50	78.69	3.15
2b	4.02	0.27	10.42	0.26	14.45	0.49	18.13	2.82
2c	-	-	0.34	0.04	1.08	0.03	3.17	0.43
3a	97.06	0.22	94.85	0.05	92.51	0.23	90.51	1.00
3b	2.93	0.22	4.96	0.09	7.19	0.12	9.02	0.95
3c	-	-	0.18	0.04	0.29	0.13	0.47	0.06
4a	93.09	0.29	85.55	0.20	79.93	1.61	74.12	0.85
4b	6.90	0.29	14.45	0.20	16.46	1.56	20.23	1.53
4c	-	-	-	-	3.60	1.45	5.65	0.80
5a	96.78	0.11	89.35	0.34	83.29	0.73	78.56	0.67
5b	3.21	0.11	9.75	0.26	13.75	0.37	17.62	0.59
5c	-	-	0.88	0.22	2.32	0.38	3.82	0.19
6a	95.95	0.31	92.75	0.16	90.03	0.44	88.14	0.68
6b	4.04	0.31	7.06	0.12	9.16	0.35	10.22	0.49
6c	-	-	0.17	0.04	0.8	0.17	1.64	0.21
7a	95.53	0.35	84.62	2.98	81.09	0.24	75.72	1.45
7b	4.23	0.36	12.67	1.09	13.67	0.20	13.95	1.20
7c	-	-	1.50	1.55	3.68	0.07	8.19	0.72
7_int1	-	-	0.20	0.22	0.47	0.05	0.919	0.30
7_int2	0.24	0.07	0.99	0.12	1.07	0.04	1.21	0.15
8a	94.78	0.17	89.73	0.44	85.15	0.32	80.58	0.28
8b	5.00	0.17	9.59	0.35	11.74	0.28	11.75	0.02
8c	0.21	0.01	0.67	0.09	3.11	0.14	7.66	0.27
9a	95.61	0.68	81.13	0.85	74.02	1.82	68.72	1.77
9b	4.38	0.68	17.03	0.57	22.83	1.61	24.30	1.34
9c	-	-	1.84	0.32	3.60	0.23	6.97	0.48

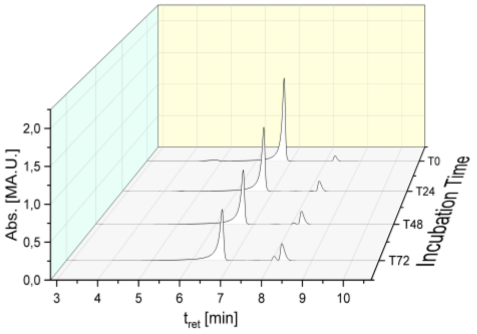

Figure S3. HPLC chromatogram of **1a** (t_{ret} = 6.05 min) over a period of 72 h in ACN/water = 50:50 (v/v) mixture. Complex **1b** appears at t_{ret} = 7.23 min and complex **1c** at t_{ret} = 7.06 min.

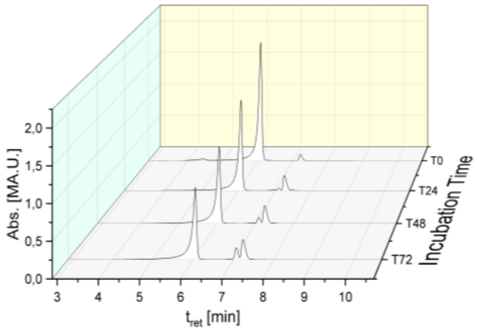

Figure S4. HPLC chromatogram of **2a** (t_{ret} = 6.74 min) over a period of 72 h in ACN/water = 50:50 (v/v) mixture. Complex **2b** appears at t_{ret} = 8.28 min and complex **2c** at t_{ret} = 8.08 min.

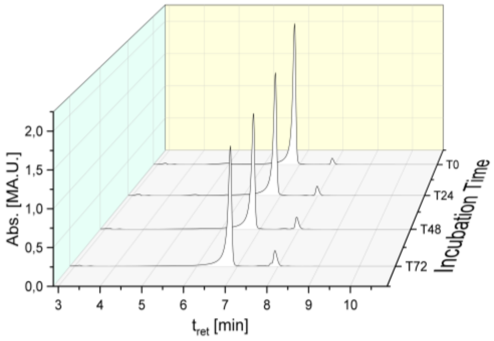

Figure S5. HPLC chromatogram of **3a** (t_{ret} = 6.81 min) over a period of 72 h in ACN/water = 50:50 (v/v) mixture. Complex **3b** appears at t_{ret} = 7.92 min and complex **3c** at t_{ret} = 7.63 min.

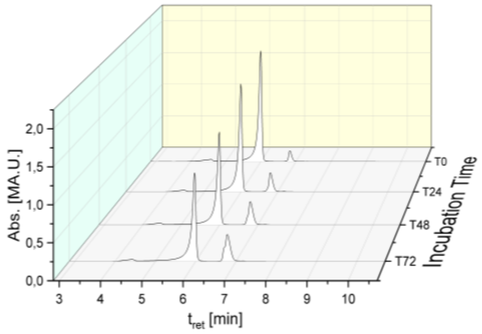

Figure S6. HPLC chromatogram of **4a** (t_{ret} = 5.96 min) over a period of 72 h in ACN/water = 50:50 (v/v) mixture. Complex **4b** appears at t_{ret} = 6.80 min.

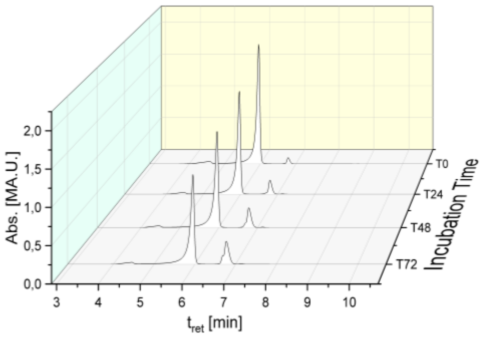

Figure S7. HPLC chromatogram of **5a** (t_{ret} = 5.94 min) over a period of 72 h in ACN/water = 50:50 (v/v) mixture. Complex **5b** appears at t_{ret} = 6.77 min.

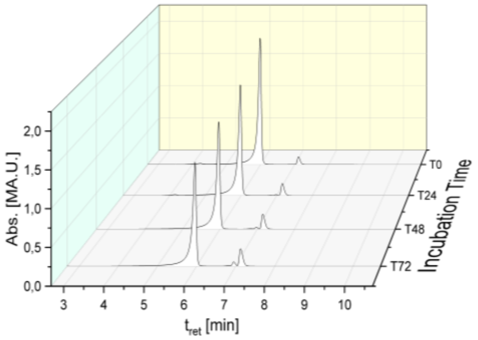

Figure S8. HPLC chromatogram of **6a** (t_{ret} = 5.95 min) over a period of 72 h in ACN/water = 50:50 (v/v) mixture. Complex **6b** appears at t_{ret} = 7.12 min and **6c** at t_{ret} = 6.94 min.

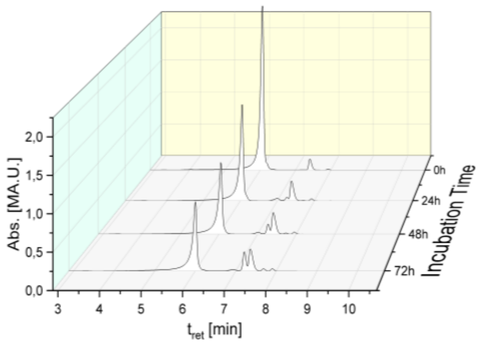

Figure S9. HPLC chromatogram of **7a** (t_{ret} = 6.08 min) over a period of 72 h in ACN/water = 50:50 (v/v) mixture. Complex **7b** appears at t_{ret} = 7.36 min and complex **7c** at t_{ret} = 7.23 min.

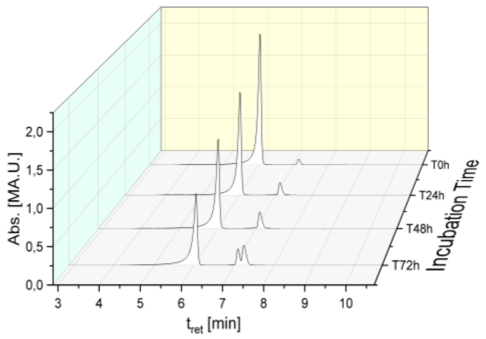

Figure S10. HPLC chromatogram of **8a** (t_{ret} = 6.01 min) over a period of 72 h in ACN/water = 50:50 (v/v) mixture. Complex **8b** appears at t_{ret} = 7.24 min and complex **8c** at t_{ret} = 7.09 min.

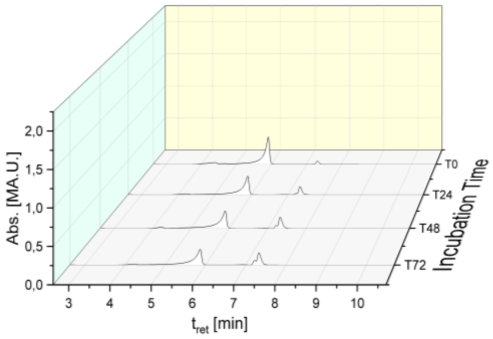

Figure S11. HPLC chromatogram of **9a** (t_{ret} = 5.83 min) over a period of 72 h in ACN/water = 50:50 (v/v) mixture. Complex **9b** appears at t_{ret} = 7.31 min and complex **9c** at t_{ret} = 7.49 min.

4. Dependence on the Solvent

Table S2. Average values (AD [%]) and standard deviation (SD [%]) of 7a (and its degradation products 7b and 7c) during incubation in different pure solvents or related 50% water mixtures at rt, investigated over a period of 72 h by HPLC.

		t = 0 h		*t = 24 h*		*t = 48 h*		*t = 72 h*	
	**Entry**	AD [%]	SD [%]	AD [%]	SD [%]	AD [%]	SD [%]	AD [%]	SD [%]
ACN (100%)	**7a**	97.15	1.37	96.35	1.39	97.05	0.05	96.97	0.25
	**7b**	2.75	1.24	3.42	1.30	2.76	0.04	2.85	0.24
	**7c**	-	-	-	-	-	-	-	-
ACN (50%)	**7a**	95.53	0.35	46.98	4.23	39.84	3.97	34.35	2.10
	**7b**	4.23	0.36	7.10	1.44	6.72	0.73	6.31	0.41
	**7c**	-	-	0.90	1.04	1.80	0.14	3.72	0.45
	**7_int1**	0.23	0.07	0.11	0.15	0.23	0.02	0.42	0.15
	**7_int2**	-	-	0.56	0.13	0.52	0.03	0.55	0.08
DMF (100%)	**7a**	94.85	0.17	93.58	0.11	92.38	0.07	92.18	0.03
	**7b**	4.85	0.09	5.94	0.06	7.05	0.03	7.29	0.04
	**7c**	-	-	-	-	-	-	-	-
	**7_int2**	0.29	0.08	0.39	0.02	0.45	0.02	0.40	0.07
DMF (50%)	**7a**	94.93	0.49	21.32	0.52	10.59	2.2	8.41	0.33
	**7b**	4.75	0.49	9.39	0.22	9.73	0.12	8.67	2.02
	**7c**	-	-	0.13	0.02	0.18	0.01	0.15	0.03
	**7_int2**	0.31	0.01	0.64	0.00	0.66	0.06	0.70	0.40
DMSO (100%)	**7a**	95.46	0.03	94.63	0.37	94.07	0.03	93.77	0.02
	**7b**	4.25	0.05	5.00	0.37	5.56	0.02	5.85	0.02
	**7c**	-	-	-	-	-	-	-	-
	**7_int2**	0.28	0.02	0.37	0.01	0.36	0.02	0.38	0.04
DMSO (50%)	**7a**	80.44	0.22	44.04	5.94	1.06	0.07	1.03	0.11
	**7b**	18.47	0.28	20.67	1.63	22.95	0.60	23.24	0.73
	**7c**	0.31	0.02	0.55	0.06	0.63	0.12	0.63	0.07
	**7_int2**	0.77	0.05	0.61	0.08	0.67	0.10	0.66	0.15
EtOH (100%)	**7a**	94.77	0.02	94.54	0.03	94.11	0.02	93.58	0.11
	**7b**	4.90	0.03	5.12	0.04	5.54	0.03	6.01	0.06
	**7c**	-	-	-	-	-	-	-	-
	**7_int2**	0.32	0.00	0.34	0.01	0.34	0.02	0.40	0.05
EtOH (50%)	**7a**	94.19	0.09	24.93	2.55	18.46	0.62	16.44	2.20
	**7b**	5.46	0.13	7.96	0.61	8.99	0.25	9.51	0.19
	**7c**	-	-	-	-	-	-	-	-
	**7_int2**	0.34	0.04	0.40	0.05	0.51	0.04	0.59	0.01
MeOH (100%)	**7a**	97.04	0.06	95.89	0.71	95.01	0.23	93.65	0.36
	**7b**	2.95	0.06	3.88	0.57	4.67	0.24	5.57	0.26
	**7c**	-	-	-	-	-	-	-	-
	**7_int2**	-	-	0.28	0.01	0.31	0.05	0.34	0.05
MeOH (50%)	**7a**	82.24	0.39	1.12	0.03	1.14	0.08	1.23	0.06
	**7b**	16.91	0.39	17.26	1.31	16.93	2.41	17.99	1.03
	**7c**	0.21	0.01	0.25	0.02	0.27	0.01	0.23	0.08
	**7_int2**	0.62	0.06	0.38	0.25	0.54	0.02	0.62	0.03

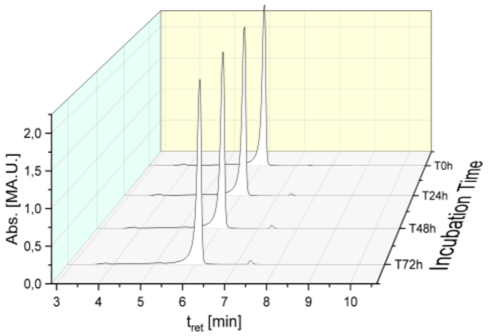

Figure S12. HPLC chromatograms after 0 h, 24 h, 48 h, 72 h of **7a** in 100% ACN during the incubation for 72 h at rt.

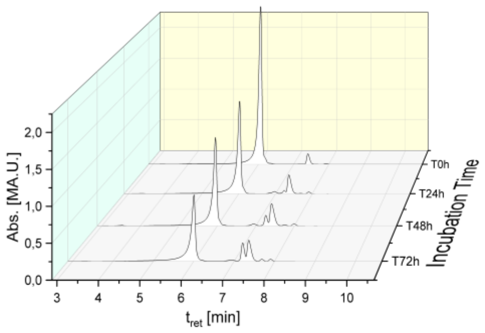

Figure S13. HPLC chromatograms after 0 h, 24 h, 48 h, 72 h of **7a** in ACN/water (50:50, v/v) during the incubation for 72 h at rt.

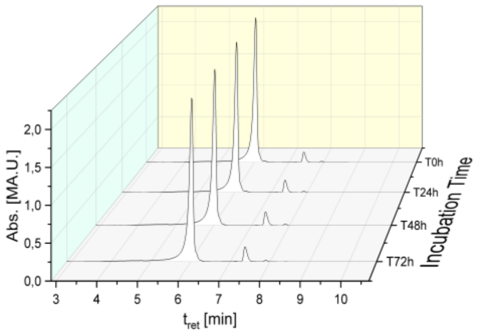

Figure S14. HPLC chromatograms after 0 h, 24 h, 48 h, 72 h of **7a** in 100% DMF during the incubation for 72 h at rt.

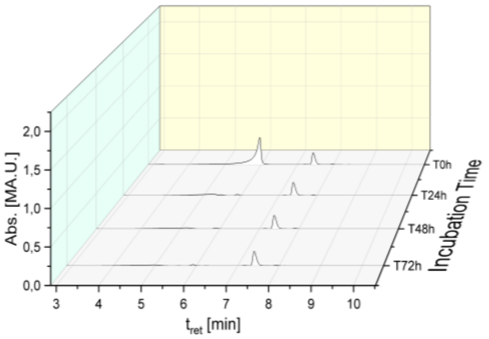

Figure S15. HPLC chromatograms after 0 h, 24 h, 48 h, 72 h of **7a** in DMF/water (50:50, v/v) during the incubation for 72 h at rt.

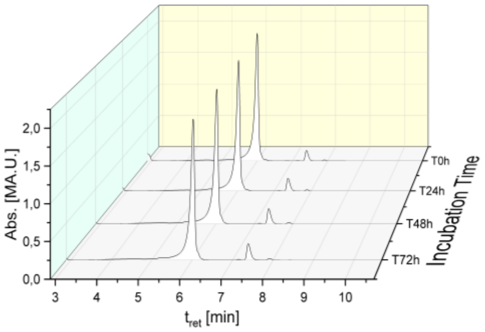

Figure S16. HPLC chromatograms after 0 h, 24 h, 48 h, 72 h of **7a** in 100% DMSO during the incubation for 72 h at rt.

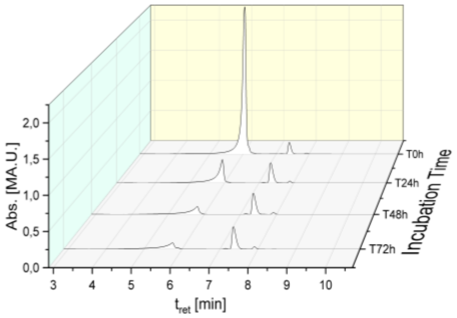

Figure S17. HPLC chromatograms after 0 h, 24 h, 48 h, 72 h of **7a** in DMSO/water (50:50, v/v) during the incubation for 72 h at rt.

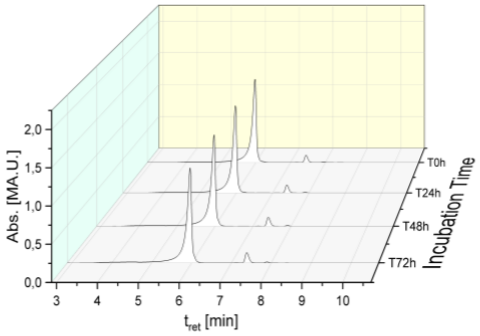

Figure S18. HPLC chromatograms after 0 h, 24 h, 48 h, 72 h of **7a** in 100% EtOH during the incubation for 72 h at rt.

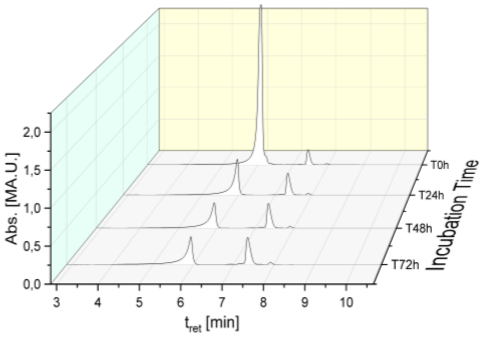

Figure S19. HPLC chromatograms after 0 h, 24 h, 48 h, 72 h of **7a** in EtOH/water (50:50, v/v) during the incubation for 72 h at rt.

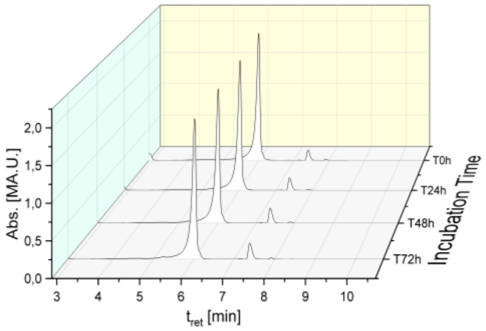

Figure S20. HPLC chromatograms after 0 h, 24 h, 48 h, 72 h of **7a** in MeOH during the incubation for 72 h at rt.

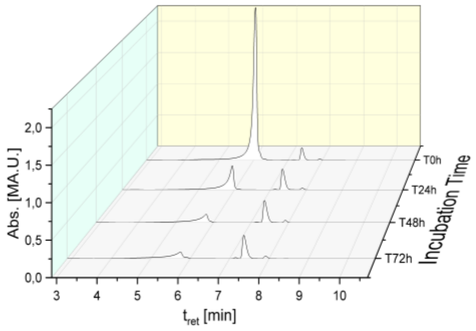

Figure S21. HPLC chromatograms after 0 h, 24 h, 48 h, 72 h of **7a** in MeOH/water (50:50, v/v) during the incubation for 72 h at rt.

5. Concentration Dependence

Table S3. Average values (AD [%]) and standard deviation (SD [%]) of 8a (and its degradation products 7b and 7c) during incubation at various concentrations (2 mM, 1 mM and 0.5 mM) in 50% water mixtures at rt, investigated over a period of 72 h by HPLC.

		t = 0 h		*t = 24 h*		*t = 48 h*		*t = 72 h*	
	**Entry**	**AD [%]**	**SD[%]**	**AD [%]**	**SD [%]**	**AD [%]**	**SD [%]**	**AD [%]**	**SD[%]**
2 mM	**8a**	94.92	0.04	90.75	1.16	82.04	0.11	76.17	0.16
	**8b**	4.97	0.04	8.41	0.80	13.12	0.14	15.42	0.16
	**8c**	0.11	0.02	0.84	0.35	4.84	0.09	8.42	0.03
1 mM	**8a**	94.78	0.17	89.73	0.44	85.15	0.32	80.58	0.28
	**8b**	5.00	0.17	9.59	0.35	11.74	0.28	11.74	0.02
	**8c**	0.21	0.01	0.67	0.09	3.11	0.14	7.66	0.27
0.5 mM	**8a**	97.38	0.12	93.87	0.28	89.64	0.48	86.27	0.24
	**8b**	2.55	0.11	5.85	0.28	9.12	0.42	8.65	0.25
	**8c**	0.04	0.02	0.26	0.01	1.23	0.07	5.07	0.17

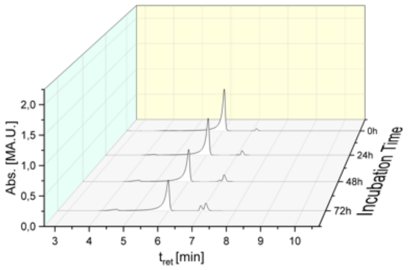

Figure S22. HPLC chromatograms of **8a** at a concentration of 0.5 mM in ACN/water (50:50, v/v) mixture after 0 h, 24 h, 48 h, and 72 h of incubation at rt.

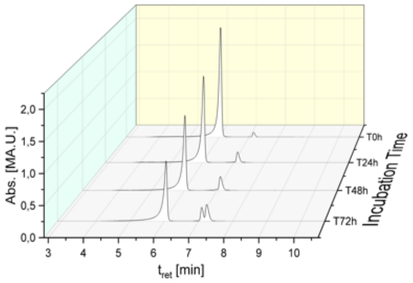

Figure S23. HPLC chromatograms of **8a** at a concentration of 1 mM in ACN/water (50:50, v/v) mixture after 0 h, 24 h, 48 h, and 72 h of incubation at rt.

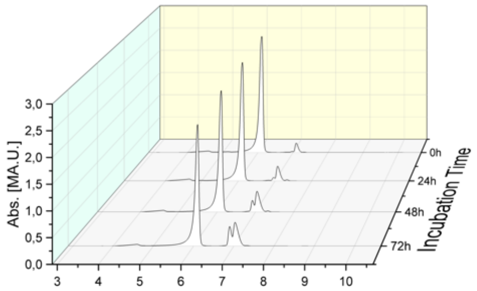

Figure S24. HPLC chromatograms of **8a** at a concentration of 2 mM in ACN/water (50:50, v/v) mixture after 0 h, 24 h, 48 h, and 72 h of incubation at rt.

6. Effect of KCl, KBr, KI or KOH Addition

Table S4. Stability of 7a (1 mM) in ACN/water (50:50, v/v) solution upon addition 20 eq. of the respective salt. Samples were incubated at rt and measured each 24 h over a period of 72 h. Average values (AD [%]) and standard deviation (SD [%]) are given.

		t = 0 h		*t = 24 h*		*t = 48 h*		*t = 72 h*	
	**Entry**	**AD [%]**	**SD [%]**	**AD [%]**	**SD [%]**	**AD [%]**	**SD [%]**	**AD [%]**	**SD [%]**
KBr	**7a**	92.31	0.56	91.30	1.02	91.50	0.16	91.07	0.24
	**7b**	7.16	0.53	8.09	0.95	7.85	0.15	8.26	0.16
	**7c**	-	-	-	-	-	-	-	-
	**7_int2**	0.53	0.05	0.60	0.06	0.65	0.01	0.67	0.09
KI	**7a**	-	-	-	-	-	-	-	-
	**7b**	25.31	7.21	30.75	0.73	29.86	1.71	30.42	0.79
	**7c**	-	-	-	-	-	-	-	-
	**7d**	72.97	7.77	67.39	0.80	68.15	1.51	67.51	0.83
	**7_int2**	1.72	0.58	1.87	0.18	1.98	0.20	2.07	0.20
KCl	**7a**	34.12	4.34	32.63	3.32	29.70	0.07	27.96	0.36
	**7b**	4.68	0.12	5.07	0.23	6.15	0.33	7.81	0.25
	**7c**	-	-	-	-	-	-	-	-
	**7e**	60.90	4.20	61.94	3.11	63.95	0.07	63.65	0.10
	**7_int2**	0.28	0.03	0.32	0.01	0.44	0.02	0.58	0.04
KOH	**7a**	89.19	1.43	88.76	0.13	88.62	0.07	88.06	0.08
	**7b**	5.48	0.06	6.47	0.05	6.78	0.02	6.96	0.03
	**7c**	0.27	0.11	0.25	0.09	0.15	0.01	0.16	0.01
	**7f**	4.67	1.30	0.48	0.01	3.92	0.03	4.29	0.05
	**7_int2**	-	-	-	-	0.52	0.01	0.52	0.01

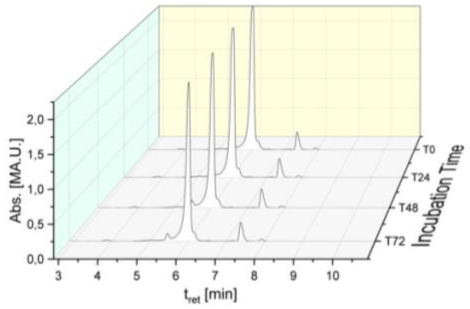

Figure S25. HPLC chromatograms of **7a** in ACN/water mixture (50:50, v/v) incubated with 20 eq. KBr after t = 0 h, 24 h, 48 h and 72 h.

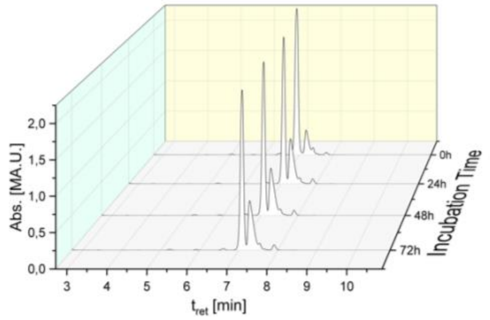

Figure S26. HPLC chromatograms of **7a** in ACN/water mixture (50:50, v/v) incubated with 20 eq. KI after t = 0 h, 24 h, 48 h and 72 h.

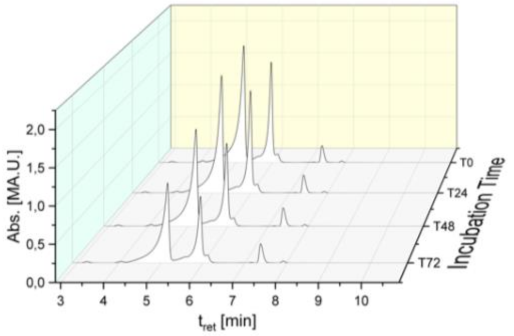

Figure S27. HPLC chromatograms of **7a** in ACN/water mixture (50:50, v/v) incubated with 20 eq. KCl after t = 0 h, 24 h, 48 h and 72 h.

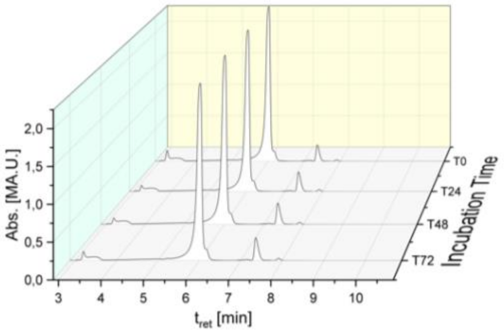

Figure S28. HPLC chromatograms of **7a** in ACN/water mixture (50:50, v/v) incubated with 20 eq. KOH after t = 0 h, 24 h, 48 h and 72 h.

Table S5. Stability of 8a (1 mM) in ACN/water (50:50, v/v) solution upon addition 20 eq. of the respective salt. Samples were incubated at rt and measured each 24 h over a period of 72 h. Average values (AD [%]) and standard deviation (SD [%]) are given.

		t = 0 h		*t = 24 h*		*t = 48 h*		*t = 72 h*	
	**Entry**	**AD [%]**	**SD [%]**	**AD [%]**	**SD [%]**	**AD [%]**	**SD [%]**	**AD [%]**	**SD [%]**
KBr	**8a**	97.76	0.05	97.61	0.58	98.29	1.13	97.53	0.33
	**8b**	2.31	0.12	2.31	0.12	2.27	0.01	2.46	00.33
	**8c**	-	-	-	-	-	-	-	-
	**8a**	-	-	-	-	-	-	-	-
KI	**8b**	17.52	0.51	25.51	1.93	23.24	5.21	23.05	0.97
	**8c**	-	-	-	-	-	-	-	-
	**8d**	82.48	0.51	74.49	1.93	76.75	5.21	76.49	0.97
	**8a**	79.67	0.37	79.16	0.37	78.42	0.42	77.56	0.53
	**8b**	2.20	0.11	3.21	0.03	5.03	0.45	6.39	0.82
KCl	**8c**	-	-	-	-	-	-	0.80	0.19
	**8e**	18.12	0.41	17.62	0.36	16.54	0.20	15.44	0.35
	**8a**	94.98	0.02	83.76	0.63	78.16	0.47	73.18	0.17
	**8b**	3.06	0.06	10.55	0.12	13.36	0.15	15.86	0.15
	**8c**	-	-	3.78	0.12	6.33	0.17	8.95	0.17
KOH	**8f**	1.57	1.07	1.90	0.50	2.14	0.14	2.00	0.04

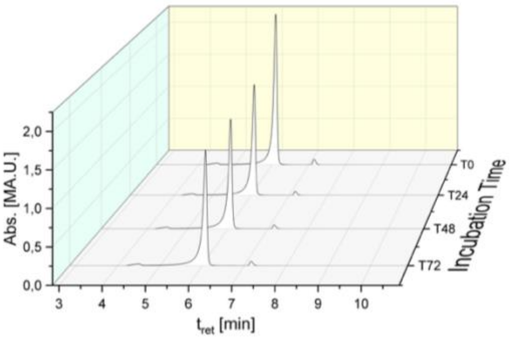

Figure S29. HPLC chromatograms of **8a** in ACN/water mixture (50:50, v/v) incubated with 20 eq. KBr after t = 0 h, 24 h, 48 h and 72 h.

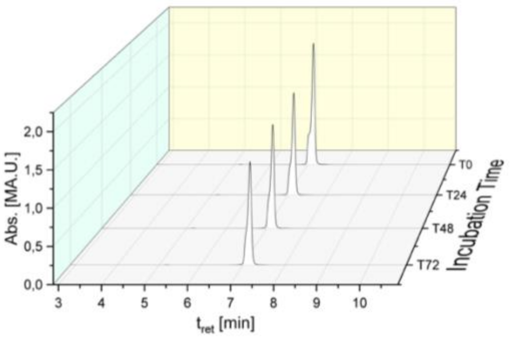

Figure S30. HPLC chromatograms of **8a** in ACN/water mixture (50:50, v/v) incubated with 20 eq. KI after t = 0 h, 24 h, 48 h and 72 h.

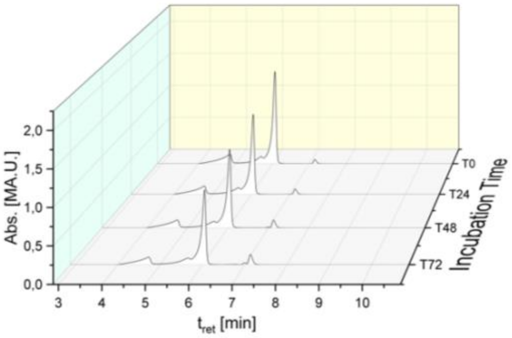

Figure S31. HPLC chromatograms of **8a** in ACN/water mixture (50:50, v/v) incubated with 20 eq. KCl after t = 0 h, 24 h, 48 h and 72 h.

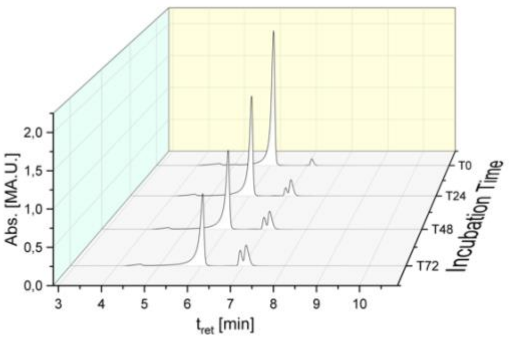

Figure S32. HPLC chromatograms of **8a** in ACN/water mixture (50:50, v/v) incubated with 20 eq. KOH after t = 0 h, 24 h, 48 h and 72 h.

7. Dependence on the Temperature

Table S6. Stability of 8a (1 mM) in ACN/water (50:50, v/v) solution at various temperatures (4°C, 22°C, 37°C, 50°C, 80°C). Samples were incubated for 72 h and the content of 8a and its degradation products 8b and 8c were analyzed by HPLC after 0 h, 24 h, 48 h, and 72 h. Average values (AD [%]) and standard deviation (SD [%]) are given.

		t = 0 h		*t = 24 h*		*t = 48 h*		*t = 72 h*	
	**Entry**	**AD[%]**	**SD [%]**	**AD [%]**	**SD [%]**	**AD [%]**	**SD [%]**	**AD [%]**	**SD [%]**
4°C	**8a**	97.23	0.10	89.92	0.73	88.84	1.15	84.69	0.36
	**8b**	2.76	0.10	9.44	0.60	9.68	0.92	12.96	0.40
	**8c**	-	-	0.64	0.17	1.47	0.37	2.33	0.09
22°C	**8a**	94.78	0.17	89.73	0.44	85.15	0.32	80.58	0.28
	**8b**	5.00	0.17	9.59	0.35	11.74	0.28	11.74	0.02
	**8c**	0.21	0.01	0.67	0.09	3.11	0.14	7.66	0.27
37°C	**8a**	97.11	0.05	82.37	1.17	71.86	0.64	59.77	0.92
	**8b**	2.89	0.05	14.31	0.76	20.70	1.20	27.89	1.62
	**8c**	-	-	3.32	1.85	7.44	1.79	12.33	2.55
50°C	**8a**	96.88	0.14	76.15	0.05	65.36	0.64	65.20	0.71
	**8b**	3.11	0.14	19.65	0.28	24.95	1.30	25.94	1.30
	**8c**	-	-	4.20	0.31	9.68	1.90	8.85	2.01
80°C	**8a**	95.40	0.37	68.51	1.21	63.66	0.09	57.84	0.14
	**8b**	4.41	0.36	27.34	0.55	36.34	0.09	42.15	0.14
	**8c**	0.19	0.06	4.14	1.76	-	-	-	-

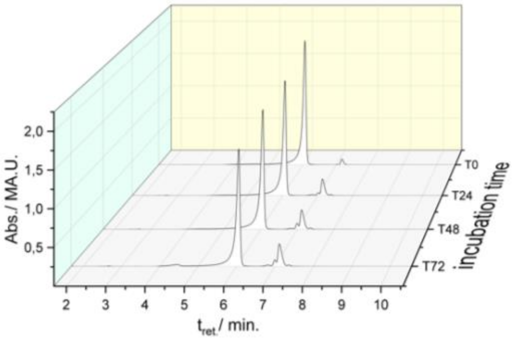

Figure S33. HPLC chromatograms of **8a** in ACN/water mixture (50:50, v/v) incubated at 4°C over a period of 72 h.

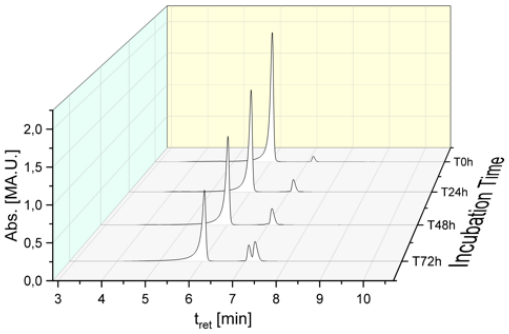

Figure S34. HPLC chromatograms of **8a** in ACN/water mixture (50:50, v/v) incubated at 22°C over a period of 72 h.

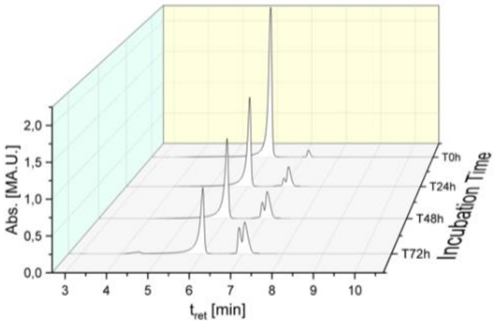

Figure S35. HPLC chromatograms of **8a** in ACN/water mixture (50:50, v/v) incubated at 37°C over a period of 72 h.

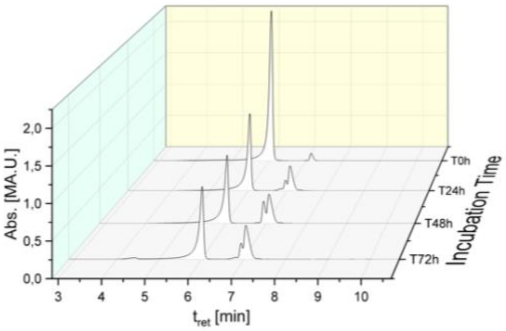

Figure S36. HPLC chromatograms of **8a** in ACN/water mixture (50:50, v/v) incubated at 50°C over a period of 72 h.

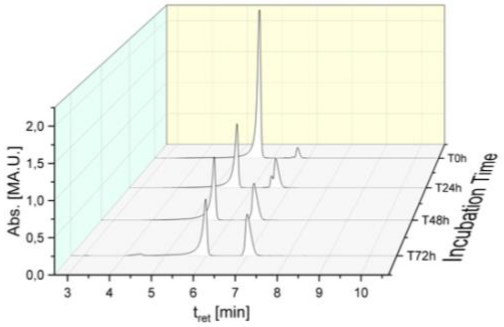

Figure S37. HPLC chromatograms of **8a** in ACN/water mixture (50:50, v/v) incubated at 80°C over a period of 72 h.

8. Stability in 0.9% NaCl

Table S7. Stability of 7a, 7b, 7c (1 mM each) in ACN/water (50:50, v/v) solution upon addition 0.9% NaCl. Samples were incubated at rt and measured each 24 h over a period of 72 h. Average values (AD [%]) and standard deviation (SD [%]) are given.

		t = 0 h		*t = 24 h*		*t = 48 h*		*t = 72 h*	
	**Entry**	**AD [%]**	**SD [%]**	**AD [%]**	**SD [%]**	**AD [%]**	**SD [%]**	**AD [%]**	**SD [%]**
7a + 0.9%NaCl	**7a**	58.06	3.20	53.94	0.41	54.44	0.22	53.15	0.58
	**7e**	34.27	2.79	37.18	0.16	35.33	0.18	33.46	0.51
	**7b**	7.16	0.36	8.33	0.19	9.50	0.07	12.35	0.06
	**7c**	-	-	-	-	-	-	-	-
	**7_int2**	0.50	0.06	0.54	0.05	0.71	0.01	1.03	0.03
7b + 0.9%NaCl	**7a**	-	-	-	-	-	-	-	-
	**7b**	100	0.00	100	0.00	100	0.00	100	0.00
	**7c**	-	-	-	-	-	-	-	-
7c + 0.9%NaCl	**7a**	-	-	-	-	-	-	-	-
	**7b**	-	-	-	-	-	-	-	-
	**7c**	89.70	8.91	84.82	0.22	85.06	0.56	85.37	0.52
	**7g**	15.44	0.33	15.18	0.22	14.93	0.56	14.63	0.52

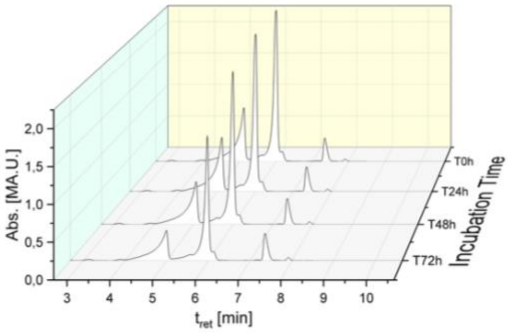

Figure S38. HPLC chromatograms of **7a** incubated in ACN/water + 0.9% NaCl (50:50, v/v) mixture.

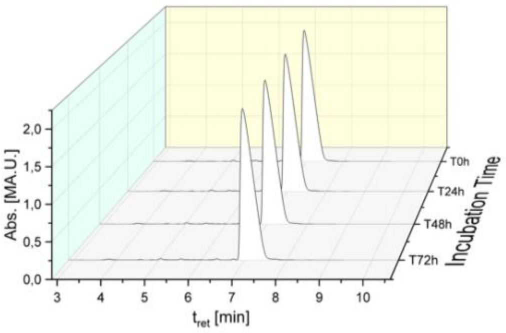

Figure S39. HPLC chromatograms of **7b** incubated in ACN/water + 0.9% NaCl (50:50, v/v) mixture.

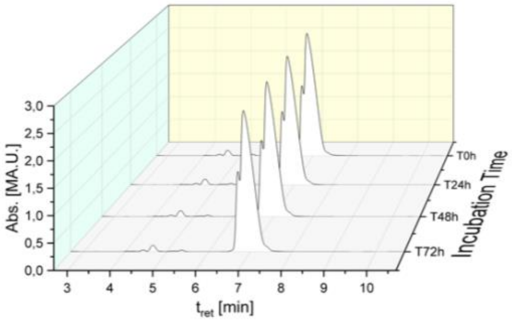

Figure S40. HPLC chromatograms of **7c** incubated in ACN/water + 0.9% NaCl (50:50, v/v) mixture.

9. UV-Spectra of Compounds **NHC ligand**, **7a**, and **7b**

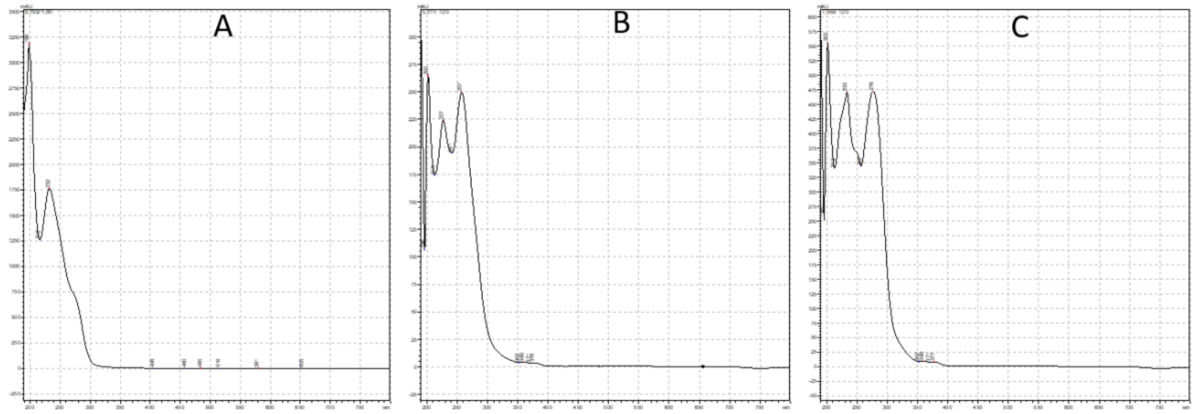

Figure S41: UV spectra of the NHC ligand (A), the bromido (NHC)gold(I) complex **7a** (B), and the bis(NHC)gold(I) derivative **7b** (C) taken in ACN/water 90/10 (v/v) at a concentration of 10^{-4} M.

References

1. Gallati, C. M.; Goetzfried, S. K.; Ausserer, M.; Sagasser, J.; Plangger, M.; Wurst, K.; Hermann, M.; Baecker, D.; Kircher, B.; Gust, R., Synthesis, characterization and biological activity of bromido[3-ethyl-4-aryl-5-(2-methoxypyridin-5-yl)-1-propyl-1,3-dihydro-2*H*-imidazol-2-ylidene]gold(I) complexes. *Dalton Trans.* **2020,** *49*, 5471-5481.
2. Gallati, C. M.; Goetzfried, S. K.; Ortmeier, A.; Sagasser, J.; Wurst, K.; Hermann, M.; Kircher, B.; Gust, R., Synthesis, characterization and biological activity of bis[3-ethyl-4-aryl-5-(2-methoxy-pyridin-5-yl)-1-propyl-1,3-dihydro-2*H*-imidazol-2-ylidene]gold(I) complexes. *Dalton Trans.* **2021**, in press.
